# Development of Singlet Oxygen Luminescence Kinetics during the Photodynamic Inactivation of Green Algae

**DOI:** 10.3390/molecules21040485

**Published:** 2016-04-13

**Authors:** Tobias Bornhütter, Judith Pohl, Christian Fischer, Irena Saltsman, Atif Mahammed, Zeev Gross, Beate Röder

**Affiliations:** 1 Department of Physics, Humboldt-Universität zu Berlin, Berlin 10099, Germany; Tobias.Bornhuetter@physik.hu-berlin.de (T.B.); Judith.Pohl@physik.hu-berlin.de (J.P.); chrisf87@live.de (C.F.); 2 Schulich Faculty of Chemistry, Technion-Israel Institute of Technology, Haifa 32000, Israel; saltsman@tx.technion.ac.il (I.S.); chatif@tx.technion.ac.il (A.M.); chr10zg@tx.technion.ac.il (Z.G.)

**Keywords:** singlet oxygen, photodynamic inactivation, phototoxicity, biofilms, corroles

## Abstract

Recent studies show the feasibility of photodynamic inactivation of green algae as a vital step towards an effective photodynamic suppression of biofilms by using functionalized surfaces. The investigation of the intrinsic mechanisms of photodynamic inactivation in green algae represents the next step in order to determine optimization parameters. The observation of singlet oxygen luminescence kinetics proved to be a very effective approach towards understanding mechanisms on a cellular level. In this study, the first two-dimensional measurement of singlet oxygen kinetics in phototrophic microorganisms on surfaces during photodynamic inactivation is presented. We established a system of reproducible algae samples on surfaces, incubated with two different cationic, antimicrobial potent photosensitizers. Fluorescence microscopy images indicate that one photosensitizer localizes inside the green algae while the other accumulates along the outer algae cell wall. A newly developed setup allows for the measurement of singlet oxygen luminescence on the green algae sample surfaces over several days. The kinetics of the singlet oxygen luminescence of both photosensitizers show different developments and a distinct change over time, corresponding with the differences in their localization as well as their photosensitization potential. While the complexity of the signal reveals a challenge for the future, this study incontrovertibly marks a crucial, inevitable step in the investigation of photodynamic inactivation of biofilms: it shows the feasibility of using the singlet oxygen luminescence kinetics to investigate photodynamic effects on surfaces and thus opens a field for numerous investigations.

## 1. Introduction

Biofilms play a major role in biofouling and the biodeterioration of construction materials. The biodeterioration of a building not only compromises its aesthetic appearance, but can also destroy cultural heritage when it comes to ancient buildings and even endanger people when the structural integrity is affected [1,2,3,4].

Established methods for the suppression or removal of biofilms have major drawbacks: mechanical removal of biofilms comes along with removal of construction material, while chemical approaches like the use of biocides pose environmental risks. This has motivated numerous efforts to find alternate ways to remove/reduce biofilms or supress their formation.

The functionalized surfaces approach appears to be a highly suitable and promising general tactic [5,6,7,8]. Most biofilms on outdoor surfaces contain phototrophic organisms, which need light to exist [9], and photocatalytic approaches have hence been adopted and displayed numerous interesting results up to now. The use of titanium dioxide in various forms stands out in that regard, though it was proven to be less effective for phototrophic organisms in field studies than under laboratory conditions. It was shown that titanium dioxide-functionalized surfaces can generate NOx species, due to electron transfer-based photocatalytic effects, and these species may act as fertilizers for the microorganisms [6,7,8,10,11,12].

Our aim is the development of functionalized surfaces, taking advantage of the photodynamic effect, as an attractive alternative to the photocatalytic effects that, e.g., titanium dioxide- functionalized surfaces, are based on. The idea of surface-immobilized photosensitizers for inhibiting the growth of biofilms is promising for several reasons. Efficient photosensitizers have been identified and developed with regard to their application in clinical photodynamic therapy (PDT) for many decades [13]. Even though the requirements for photosensitizers differ between PDT and the photodynamic inactivation (PDI) of microorganisms, a variety of photosensitizers for PDI are available [14]. They all have an intense UV/vis absorption spectrum, with quite a dominant absorption in the visible part in common. In contrast to titanium dioxide, which absorbs in the UV, surfaces functionalized with photosensitizers would use a much larger part of the Sun’s emission spectrum reaching our planet. Another advantage of photosensitizers is their low to non-existing dark toxicity, having been developed for medical use in most cases. In contrast to that, the concern regarding the toxicity of titanium dioxide- and nanoparticle-based photocatalysis applications to people and environment is raising, as the use of titanium dioxide in several fields of application is increasing [8,15,16].

Even though PDT is in widespread use today [13] and photodynamic inactivation of bacteria (PIB) is becoming an acknowledged approach [14,17,18,19,20,21], only a few examples on PDI of phototrophic organisms have been reported [22,23,24,25]. A first preliminary step towards developing photoactive surfaces using the photodynamic effect, was reported by Pohl *et al.*, who revealed the feasibility of green algae inhibition using the photodynamic effect [25].

For advancing the PDI of phototrophic microorganisms beyond a mere proof of concept, we considered that the next step should be to evaluate the utilization of direct singlet oxygen (^1^O_2_) luminescence and photosensitizer fluorescence measurements to characterize and optimize the conditions for photodynamic inactivation of green algae. Fluorescence spectroscopy in general is a powerful tool; however, it was proven in the past that the combination of fluorescence spectroscopy and the time-resolved detection of the phosphorescence of generated ^1^O_2_ is the most effective approach to investigate photodynamic inactivation processes. While the photosensitizer′s fluorescence may be analysed for deducing on its location and possible aggregation, the analysis of the ^1^O_2_ luminescence kinetics provides an insight on the microenvironment of the photosensitizer. This may be used for revealing the biochemical mechanism of the inactivation, thus allowing for optimization of the photosensitizer in terms of localisation and effective singlet oxygen generation [26,27].

## 2. Results

**Figure 1:**
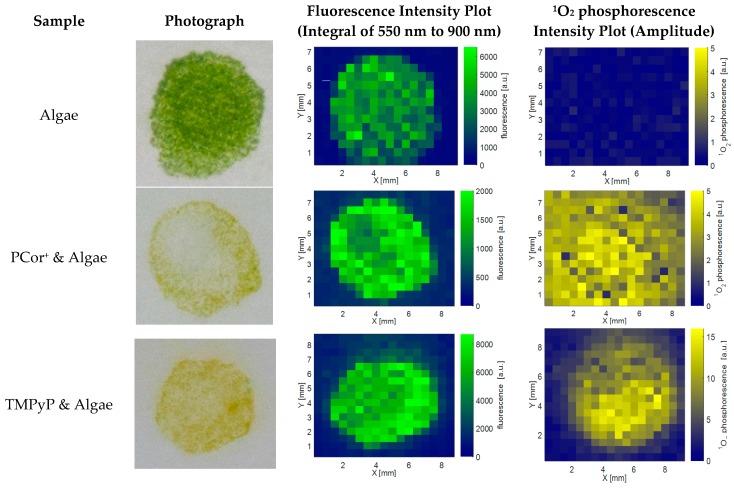
Comparison of the photographs, fluorescence intensity plots and singlet oxygen intensity plots of all samples. As expected, the algae reference does not display any ^1^O_2_ phosphorescence. The TMPyP-algae-sample shows high spatial correlation of the photograph, and both the fluorescence and ^1^O_2_ phosphorescence intensity plots. Interestingly, the ^1^O_2_ phosphorescence intensity plot of the PCor^+^-algae-sample appears to contradict the fluorescence intensity plot and the photograph.

Figure 1 shows photographs, spatial fluorescence and ^1^O_2_ phosphorescence plots of the algae reference, the PCor^+^-algae, and the TMPyP-algae samples after one day of incubation (on day 2). Photographs of the samples are presented in the first column, the spatial distribution of the fluorescence in the second and the spatial distribution of the ^1^O_2_ phosphorescence in the third column. The photographs illustrate the sample geometry and the inhomogeneity due to the sample preparation technique. The fluorescence intensity in the plot equates the integral over the spectral range of 550–900 nm. The ^1^O_2_ phosphorescence intensity shown in Figure 1 equates the fitted amplitude.

As expected, the algae reference is fluorescent, but devoid of any ^1^O_2_ phosphorescence. The TMPyP-algae sample shows spatial correlation of fluorescence and ^1^O_2_ phosphorescence even though the sharp edges of the sample that can be seen in the photograph and in the fluorescence plot appear smooth in the ^1^O_2_ phosphorescence plot. In contrast to this high spatial correlation, there is hardly any spatial correlation between the photograph and the fluorescence plot on one hand and the ^1^O_2_ phosphorescence plot on the other hand in the PCor^+^-algae sample.

**Figure 2:**
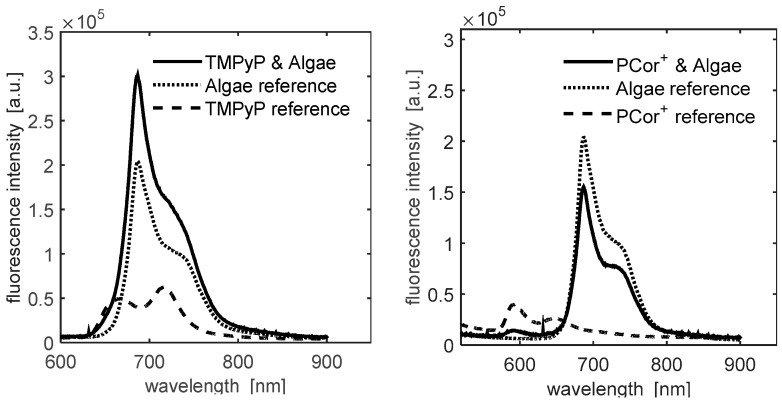
Comparison of fluorescence spectra of PCor^+^ and TMPyP with algae to photosensitizer and algae reference (fluorescence intensity not corrected for different sample geometry).

The abovementioned puzzling mismatch was resolved by recording the full fluorescence spectra of all samples on day 2. Figure 2 shows the fluorescence spectra summed up over five pixels of highest signal intensity, which reveals that: (a) the fluorescence intensity due to the algae′s photosynthesis system is much stronger than that of the photosensitizers; (b) hardly any information can be drawn from the integrated fluorescence measurements of the algae/TMPyP combination because of the almost complete overlap of the emission spectra of the components; (c) the PCor^+^′s fluorescence spectrum is located partly outside the algae emission, which may be used for its separate evaluation.

**Figure 3:**
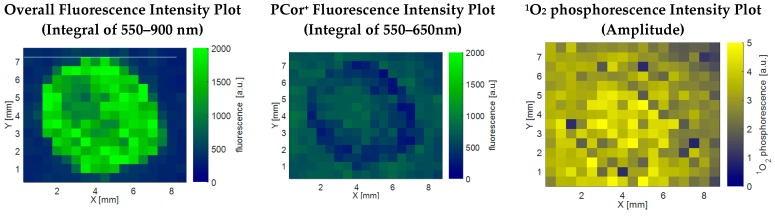
Comparison of the fluorescence intensity plots integrated over the whole recorded spectrum and the for the PCor^+^ fluorescence relevant part of the recorded spectrum and the ^1^O_2_ phosphorescence intensity plot for a PCor^+^-algae sample. In contrast to TMPyP, PCor^+^ appears to diffuse in the surrounding filter paper.

The latter hypothesis was confirmed by the results depicted in Figure 3. The left plot shows the fluorescence intensity as above, *i.e.*, integrated from 550 to 900 nm. The plot in the middle shows the fluorescence intensity integrated only over the range of 550–650 nm, so as to isolate the PCor^+^′s fluorescence. This fluorescence plot is in correlation with the ^1^O_2_ phosphorescence plot, indicating a diffusion of the photosensitizer onto the filter paper.

This evaluation of the spatial distribution of the fluorescence signal illustrates the importance of combining it with direct ^1^O_2_ phosphorescence measurements. As mentioned in the Introduction, the ^1^O_2_ kinetics provides information about the microenvironment of the photosensitizer. The most vital requirement for a detailed analysis of ^1^O_2_ kinetics is a sufficient signal strength. To check this requirement and to explain how the later shown data was obtained, an excursion to the performed data analysis is necessary.

During this study, no changes were observed between the first and second scans of each sample on each day. Therefore, the signals of both scans per day were summed up pixel by pixel for each sample. The signal for the analysis and comparison of the samples and their temporal evolution were obtained by estimating the signal intensity of each pixel, evaluating the five pixels with highest intensity and calculating the sum of the signal of these five pixels.

To facilitate the comparison of the different signals, the standard biexponential model as described in [28] was extended by additional exponential terms in order to fit the data. The standard biexponential model for ^1^O_2_ kinetics is the solution of a rate equation contemplating the generation and deactivation processes of ^1^O_2_. The necessity of extending the biexponential model depends on the sample and is discussed along with the respective results. In general, the extension of the standard biexponential model was required because it describes ^1^O_2_ kinetics in a homogeneous microenvironment, a condition that is apparently not experienced by the photosensitizers in the samples described in this study.

This approach was chosen to provide facile comparison of the signals and to visualize their development. As it is purely phenomenological for this study, no fitted parameter will be discussed here. The investigation of the applicability of this approach and the detailed analysis of the kinetics is an object of future work.

**Figure 4:**
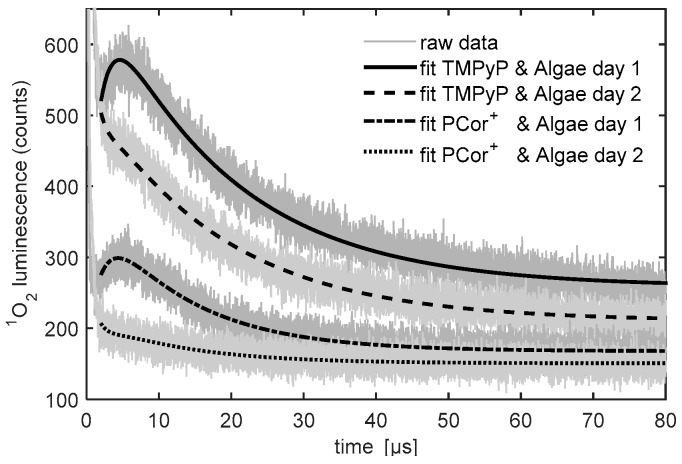
Raw data and fitted model functions.

Figure 4 shows the raw data and the fitted model functions for the first two days, obtained in the aforementioned fashion. Even though the detailed analysis of the kinetics parameters is not the subject of this article, which presents a feasibility study, changes of the ^1^O_2_ kinetics during the first two days of measurement and differences in the ^1^O_2_ kinetics between the two photosensitizers as well as references and algae samples are clearly observed.

**Figure 5:**
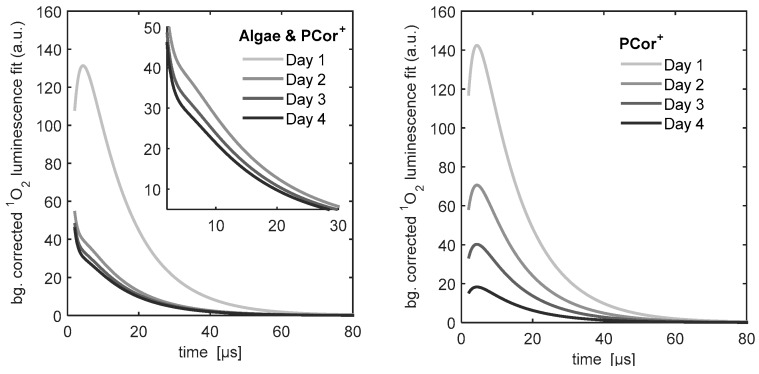
Temporal development of the singlet oxygen signals, measured on the surfaces of the PCor^+^ reference samples and the samples of PCor^+^ incubated in green algae over four days.

Figure 5 shows the ^1^O_2_ kinetics of the PCor^+^ reference sample and the PCor^+^-algae sample. The ^1^O_2_ kinetics of the reference sample could be described by the unmodified biexponential model, *i.e.*, without any extension. For the kinetics of the ^1^O_2_ kinetics of the PCor^+^-algae sample, the biexponential model had to be extended by one additional simple exponential term.

**Figure 6:**
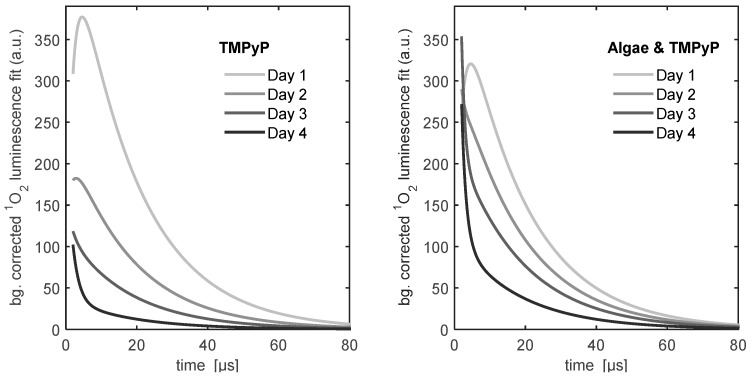
Temporal development of the singlet oxygen signals, measured on the surfaces of the TMPyP reference sample and the samples of TMPyP incubated in green algae over four days.

Figure 6 shows the ^1^O_2_ kinetics of the TMPyP reference sample and the TMPyP-algae sample. An additional simple exponential term has to be added to the biexponential model to describe the singlet oxygen luminescence kinetics, even for the TMPyP reference. The most complex kinetics were found for the TMPyP-algae sample, where two additional exponential terms had to be added to the biexponential model to fit the measured kinetic data. It must be pointed out that, as mentioned before, these fits were performed phenomenologically in order to facilitate the comparison of the data.

Moreover, measurements at 1210 nm show a weak NIR signal for all TMPyP samples, in contrast to the algae references and all PCor^+^ samples where no signal was observed. The signal decreases exponentially over several μs, corresponding to one of the additional exponential terms of the ^1^O_2_ kinetics. Due to the kinetics and the design of both, the laser and the ^1^O_2_ detection system, a measurement artefact is very unlikely. Since the signal is not significant in comparison to the here presented signals, it is not shown here.

**Figure 7:**
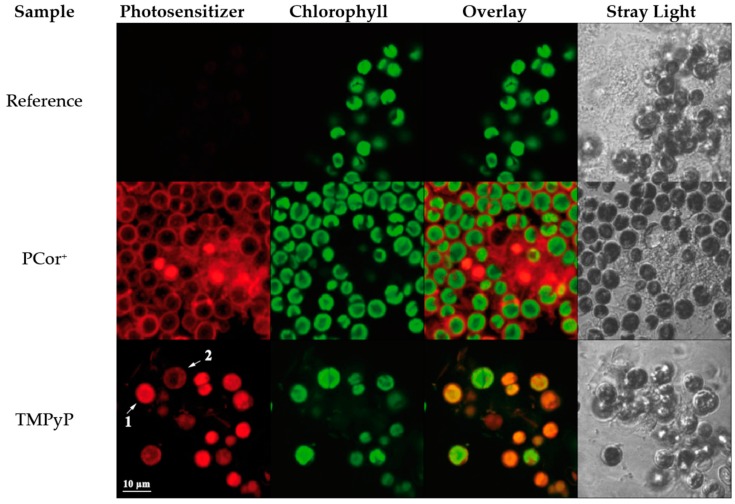
Localisation of the photosensitizer in Chlorella fusca var. vacuolata after three days of incubation. PCor^+^ appears to locate outside the algae cells while TMPyP appears to accumulate inside the algae cells.

Intra- *vs.* extra-cellular localization of the photosensitizers in the green algae after incubation was evaluated via CLSM and compared to the intrinsic fluorescence of chromophores (chlorophylls) inside the algae. In Figure 7, the resulting images of an untreated reference as well as samples incubated with either one of the two PS are shown alongside scattering images of the monitored cells. In contrast to scanning the fluorescence on macroscopic areas, CLSM imaging allows for separation of the fluorescence signals of PCor^+^ and intrinsic chlorophylls by eliminating cross talk between channels using untreated reference samples. The (red coded) fluorescence of PCor^+^ is apparent only at the periphery of the live cells (seen as circular lines of varying thickness), there is absolutely no overlap of signals attributed to intracellular chlorophyll and PCor^+^, and the few cases where PCor^+^′s fluorescence appears round may be safely attributed to dead cells. The latter conclusion may also be deduced from the scattering image where no apparent cellular structures can be found in those regions.

In contrast, fluorescence of TMPyP originates almost entirely from areas that also emit fluorescence of algae-chromophores. The distribution of the TMPyP fluorescence varies from fully filled circular areas (see Figure 7, arrow 1) to detailed structures (see Figure 7, arrow 2). The scattering image of the same sample shows cellular structures at all areas of fluorescence origin, with varying apparent viability of the cells.

## 3. Discussion

The obvious mismatch in the spatial correlation of fluorescence and ^1^O_2_ phosphorescence for the PCor^+^-algae sample in Figure 3 emphasizes once more the importance of direct ^1^O_2_ phosphorescence detection for the investigation of photodynamic inactivation processes. The spatial distribution of the ^1^O_2_ phosphorescence further indicates a diffusion of this photosensitizer into the surrounding filter paper, which is much more significant than for TMPyP. The ^1^O_2_ phosphorescence examinations reveal a nearly homogeneous allocation for the PCor^+^-algae sample, while only the smoothing of the edges indicates a diffusion of TMPyP. This kind of effect would probably go unnoticed by looking only at the spatial correlation of the fluorescence, especially for TMPyP, where it is impossible to separate the TMPyP fluorescence and algae autofluorescence.

CLSM images clearly indicate a localisation of PCor^+^ mainly onto the cell wall of the algae while TMPyP appears to accumulate inside the algae cell. With respect to future applications regarding the suppression of biofilms, photosensitizers accumulating and acting from the outside are clearly preferable.

The development of the ^1^O_2_ phosphorescence kinetics of the PCor^+^ reference shows a decrease of the signal with hardly any change in the kinetics. This indicates either a photobleaching of the photosensitizer or a change of the photosensitizer concentration on the investigated surface. Since PCor^+^ is highly water soluble and did not show any signs of photobleaching in prior studies, the possibility of a diffusion of the photosensitizer in the BBM-agar film appears much more likely.

The development of the ^1^O_2_ phosphorescence kinetics of the TMPyP reference raises more issues than that of the PCor^+^ reference. The decrease of the signal indicates a similar diffusion in the BBM-agar film. In prior experiments, TMPyP showed such diffusion into the agar substrates.

The percentage of exponential increase in the signal, developing after 4 days, is due to unobvious effects that will have to be investigated in the future. The very weak luminescence at 1210 nm, whose kinetics corresponds to one of the additional exponential terms of the ^1^O_2_ kinetics, which was measured for all TMPyP samples, indicates an autophosphorescence of TMPyP. However, this auto-phosphorescence alone obviously cannot explain the signal development.

The development of the ^1^O_2_ phosphorescence kinetics of all the algae-photosensitizer samples have two effects in common: after one day of incubation the ^1^O_2_ phosphorescence kinetics changes drastically and an exponential increase in the signal is observed over time. Since the first measurement was performed right after the sample preparation, it may safely be assumed that nearly no interaction of algae and photosensitizer took place. That explains the similarity of the measurements of the photosensitizer reference measurements and the photosensitizer-algae samples on day one.

After one day of incubation, the ^1^O_2_ phosphorescence kinetics of the PCor^+^-algae sample reaches a nearly stable condition for the rest of the measurement period. Only the amplitude decreases slightly. A correlation of this decrease with cell viability is a reasonable assumption, which however must be further validated by viability assays. This behaviour is in line with the indications for localisation of the PCor^+^ on the outside of the cell wall, since this kind of process should take place in a time period of less than a day. In this context, the development of the ^1^O_2_ phosphorescence kinetics of the TMPyP-algae samples also correlates with the assumed localisation of the TMPyP inside the algae, as intracellular uptake of this and related porphyrins has been shown to require several days. In this study, no ^1^O_2_ phosphorescence signal was measured on green algae reference samples, despite being formed during photosynthesis. This is due to the abundance of naturally occurring molecules like carotenoids inside phototrophic organisms, which act as quenchers for naturally generated amounts of ^1^O_2_. As shown in [25], this does not hinder the photodynamic inactivation of green algae. It was shown herein for the first time that the measurement of ^1^O_2_ phosphorescence kinetics on surfaces containing phototrophic organisms is possible. Even though the kinetics are complex, a distinct development of the kinetics over the four days of measurement that correlates with the localization of the photosensitizer can be observed.

## 4. Materials and Methods

**Figure 8:**
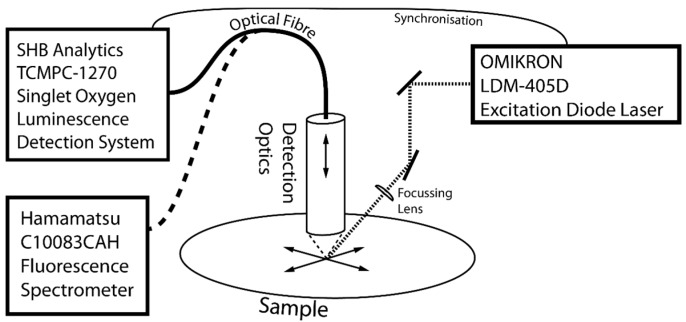
Scheme of ^1^O_2_ scanning setup.

An experimental setup was constructed for obtaining reproducible scans of luminescence on sample surfaces (Figure 8). It consists of an LDM-405D excitation laser (Omikron-Laserage, Rodgau-Dudenhofen, Germany) a cross table allowing one to scan a sample in the X and Y directions, a detection optics system coupling the excited luminescence into a fibre, movable in the Z direction, a TCMP-1270 Singlet Oxygen Luminescence Detection System by SHB Analytics (Berlin, Germany) and a C10083CAH Fluorescence Spectrometer by Hamamatsu (Hamamatsu, Shizuoka, Japan).

The TCMPC-1270 Singlet Oxygen Detection System allows for the time-resolved measurement of NIR-luminescence with highest sensitivity. Using a Hamamtsu H10330 photomultiplier tube, TCMPC electronics with time frames of 80 μs, 160 μs and 320 μs, and optical band path filters with centre wavelengths of 1270 nm and 1210 nm (both ± 15 nm), it is optimized for the detection of very weak singlet oxygen luminescence signals. The Omikron LDM-405D laser is a 2 W diode laser of the TA Deepstar series with a wavelength of 405 nm. With a modulation speed of up to 150 MHz it is highly suitable as excitation laser in combination with the TCMPC-1270. The laser is PC controlled and modulated by the TCMPC-1270. The laser beam is focused to a spot of *ca.* 150 μm. The size of the laser spot is a good approximation for a lower boundary of the spatial resolution. The scanning motion is realized with custom build mechanics and software-controlled stepping motors.

The fluorescence detection can be realized by either coupling the detection fibre directly in the Hamamatsu C10083CAH fluorescence spectrometer or by using a dichroic mirror with a longpass cutoff wavelength of 1000 nm to detect fluorescence and ^1^O_2_ phosphorescence simultaneously. To avoid the signal losses due to the dichroic mirror, in the later presented data, fluorescence and ^1^O_2_ phosphorescence were measured sequentially.

A strain of green algae *Chlorella fusca* var*. vacuolata* (SAG 211-8b) was used as model organism for measurement of ^1^O_2_ phosphorescence in phototrophic organisms. Suspension cultures of the algae were inoculated 3 days prior to experiments and grown at room temperature with shaking on a rotary shaker at 250 rpm in Bold's Basal Medium (according to [29]). Illumination of the algal cultures during cultivation was realized with a daylight bulb (Photographic Lamp, 5400 K, Realm Industrial GmbH, Berlin, Germany) in a day–night cycle of 12 h:12 h. All suspension cultures were provided with fresh medium directly before the start of any of the experiments.

**Figure 9:**
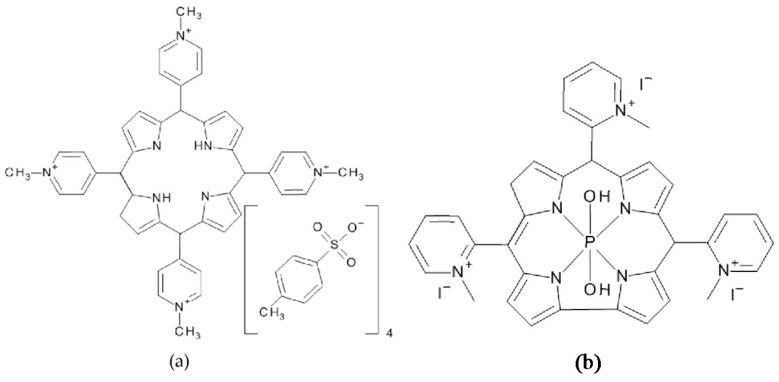
Chemical structures of the photosensitizers TMPyP and PCor^+^.

5,10,15,20-Tetrakis(1-methylpyridinium-4-yl)porphyrin tetra(*p*-toluenesulfonate) (TMPyP) and 5,10,15-tris-(1-methylpyridinium-2-yl)corrolato-(*trans*-dihydroxo)phosphorus(V) (PCor^+^), the corrole-based photosensitizer reported in [17,25], were used as photosensitizers (Figure 9). TMPyP (CAS 36951-72-1) was purchased from Sigma-Aldrich Chemie GmbH (Munich, Germany), while the tricationic metallocorrole PCor^+^ was synthesized according to [17].

For obtaining reproducible sample surfaces, algal cultures for the experiments were grown on filter paper (∅90 mm) on Bold′s Basal Medium (BBM) agar plates. To this end, 200 μL of algal suspension cultures with an initial cell density of 7 × 10^6^ mL^−1^ were placed on a circular area with a diameter of 5–7 mm. Photosensitizers were added to the cultures prior to placement on the filter paper in a concentration of 5 μmol/L. Samples of photosensitizer without green algae and green algae without photosensitizer were prepared as positive and negative controls for the singlet oxygen luminescence measurements, respectively.

The samples were covered with a fused silica window. This allows a high reproducibility of the singlet oxygen luminescence scan and prevents the samples from drying out. The samples were exposed to the 12 h day-night cycle using the 5400 K 20 W daylight bulbs for 4 days.

The singlet oxygen luminescence was scanned twice a day on an area of 8 mm by 6 mm with 1 mm pixel width in each direction. At each pixel the NIR luminescence signal was measured for 20 s using the 1270 nm bandwidth filter and an excitation energy of 0.5 μJ. On days 1 and 4, additional scans of the NIR luminescence at 1210 nm were performed under the same conditions.

To get information about the localisation of the photosensitizer in the green algae, incubated samples were examined by confocal laser scanning microscopy (CLSM) using a Fluo-ViewTM FV1000 (Olympus, Hamburg, Germany) after 3 days of incubation. Samples of *Chlorella fusca* var. *vacuolata* were grown on 2 cm × 2 cm plastic slides for microscopy (Carl Roth GmbH, Karlsruhe, Germany) incubated in suspension cultures with initial cell density of 7 × 10^6^ mL^−1^. The photosensitizers were applied to the cultures at a concentration of 5 μmol/L. After 3 days of incubation, the plastic slides were rinsed with BBM and the remaining biofilms and precipitate was examined using a 405 nm laser for excitation. The fluorescence from the samples was detected on two channels: between 570 and 640 nm and above 640 nm using a bandpass filter. To avoid fluorescence crosstalk between those channels, excitation intensity and detector amplification were adjusted using untreated samples of the algae, until the intrinsic fluorescence signal is detected only in the second channel.

## Abbreviations

The following abbreviations are used in this manuscript:

BBM	Bold′s Basal Medium
PDT	Photodynamic Therapy
PDI	Photodynamic Inactivation
PIB	Photodynamic Inactivation of Bacteria
^1^O_2_	Singlet Oxygen
TMPyP	5,10,15,20-Tetrakis(1-methylpyridinium-4-yl)porphyrin tetra(*p*-toluenesulfonate)
PCor^+^	5,10,15-tris-(1-methylpyridinium-2-yl)corrolato-(*trans*-dihydroxo)phosphorus(V)
CLSM	Confocal Laser Scanning Spectroscopy

## References

[1] Warscheid T., Braams J. (2000). Biodeterioration of stone: A review. Int. Biodeterior. Biodegrad..

[2] Miller A.Z., Sanmartín P., Pereira-Pardo L., Dionísio A., Saiz-Jimenez C., Macedo M.F., Prieto B. (2012). Bioreceptivity of building stones: A review. Sci. Total Environ..

[3] Turick C.E., Berry C.J. (2016). Review of concrete biodeterioration in relation to nuclear waste. J. Environ. Radioact..

[4] Häubner N., Schumann R., Karsten U. (2006). Aeroterrestrial microalgae growing in biofilms on facades-response to temperature and water stress. Microb. Ecol..

[5] Kochkodan V., Hilal N. (2015). A comprehensive review on surface modified polymer membranes for biofouling mitigation. Desalination.

[6] Graziani L., Quagliarini E., Osimani A., Aquilanti L., Clementi F., Yéprémian C., Lariccia V., Amoroso S., D′Orazio M. (2013). Evaluation of inhibitory effect of TiO_2_ nanocoatings against microalgal growth on clay brick façades under weak UV exposure conditions. Build. Environ..

[7] MacMullen J., Zhang Z., Dhakal H.N., Radulovic J., Karabela A., Tozzi G., Hannant S., Alshehri M.A., Buhé V., Herodotou C. (2014). Silver nanoparticulate enhanced aqueous silane/siloxane exterior facade emulsions and their efficacy against algae and cyanobacteria biofouling. Int. Biodeterior. Biodegrad..

[8] Byrne J., Dunlop P., Hamilton J., Fernández-Ibáñez P., Polo-López I., Sharma P., Vennard A. (2015). A Review of Heterogeneous Photocatalysis for Water and Surface Disinfection. Molecules.

[9] Hallmann C., Rüdrich J., Enseleit M., Friedl T., Hoppert M. (2011). Microbial diversity on a marble monument: A case study. Environ. Earth Sci..

[10] Wang L., Zhang C., Wu F., Deng N. (2007). Photodegradation of aniline in aqueous suspensions of microalgae. J. Photochem. Photobiol. B Biol..

[11] Pacheco-Torgal F., Jalali S. (2011). Nanotechnology: Advantages and drawbacks in the field of construction and building materials. Constr. Build. Mater..

[12] Gladis F., Schumann R. (2011). Influence of material properties and photocatalysis on phototrophic growth in multi-year roof weathering. Int. Biodeterior. Biodegrad..

[13] Allison R.R., Downie G.H., Cuenca R., Hu X.-H., Childs C.J.H., Sibata C.H. (2004). Photosensitizers in clinical PDT. Photodiagn. Photodyn. Ther..

[14] Preuss A., Zeugner L., Hackbarth S., Faustino M.A.F., Neves M.G.P.M.S., Cavaleiro J.A.S., Roeder B. (2013). Photoinactivation of *Escherichia coli* (SURE2) without intracellular uptake of the photosensitizer. J. Appl. Microbiol..

[15] Bogdan J., Jackowska-Tracz A., Zarzyńska J., Pławińska-Czarnak J. (2015). Chances and limitations of nanosized titanium dioxide practical application in view of its physicochemical properties. Nanoscale Res. Lett..

[16] Shi H., Magaye R., Castranova V., Zhao J. (2013). Titanium dioxide nanoparticles: A review of current toxicological data. Part. Fibre Toxicol..

[17] Preuß A., Saltsman I., Mahammed A., Pfitzner M., Goldberg I., Gross Z., Röder B. (2014). Photodynamic inactivation of mold fungi spores by newly developed charged corroles. J. Photochem. Photobiol. B Biol..

[18] Maisch T., Eichner A., Späth A., Gollmer A., König B., Regensburger J., Bäumler W. (2014). Fast and effective photodynamic inactivation of multiresistant bacteria by cationic riboflavin derivatives. PLoS ONE.

[19] Eichner A., Gollmer A., Späth A., Bäumler W., Regensburger J., König B., Maisch T. (2015). Fast and effective inactivation of Bacillus atrophaeus endospores using light-activated derivatives of vitamin B2. Photochem. Photobiol. Sci..

[20] Maisch T. (2015). Resistance in antimicrobial photodynamic inactivation of bacteria. Photochem. Photobiol. Sci..

[21] Maisch T., Baier J., Franz B., Maier M., Landthaler M., Szeimies R.-M., Bäumler W. (2007). The role of singlet oxygen and oxygen concentration in photodynamic inactivation of bacteria. Proc. Nat. Acad. Sci. USA.

[22] Drábková M., Marsálek B., Admiraal W. (2007). Photodynamic therapy against cyanobacteria. Environ. Toxicol..

[23] Young M.E., Alakomi H.-L., Fortune I., Gorbushina A.A., Krumbein W.E., Maxwell I., McCullagh C., Robertson P., Saarela M., Valero J. (2008). Development of a biocidal treatment regime to inhibit biological growths on cultural heritage: BIODAM. Environ. Geol..

[24] McCullagh C., Robertson P.K.J. (2006). Photosensitized Destruction of Chlorella vulgaris by Methylene Blue or Nuclear Fast Red Combined with Hydrogen Peroxide under Visible Light Irradiation. Environ. Sci. Technol..

[25] Pohl J., Saltsman I., Mahammed A., Gross Z., Röder B. (2015). Inhibition of green algae growth by corrole-based photosensitizers. J. Appl. Microbiol..

[26] Hackbarth S., Röder B. (2015). Singlet oxygen luminescence kinetics in a heterogeneous environment-identification of the photosensitizer localization in small unilamellar vesicles. Photochem. Photobiol. Sci..

[27] Hackbarth S., Schlothauer J., Preuss A., Röder B. (2013). Highly sensitive time resolved singlet oxygen luminescence detection using LEDs as the excitation source. Laser Phys. Lett..

[28] Schlothauer J., Röder B., Hackbarth S., Lademann J. (2010). *In vivo* detection of time-resolved singlet oxygen luminescence under PDT relevant conditions. SPIE Proc..

[29] Bischoff H.W., Bold H.C. (1963). Some Soil Algae from Enchanted Rock and Related Algal Species.

